# The impact of the SARS-CoV-2 pandemic on umbilical cord blood transplantation in Japan: insights from an interrupted time series analysis

**DOI:** 10.1038/s41409-025-02588-0

**Published:** 2025-04-17

**Authors:** Nobuhiko Nakamura, Tetsuji Morishita, Hiromi Hayashi, Motohito Okabe, Hideki Nakasone, Naoyuki Uchida, Noriko Doki, Takahiro Fukuda, Satoshi Yoshihara, Masatsugu Tanaka, Tetsuya Nishida, Yuta Hasegawa, Ken-ichi Matsuoka, Masashi Sawa, Tetsuya Eto, Makoto Onizuka, Yuta Katayama, Koji Kato, Fumihiko Ishimaru, Ken Tabuchi, Yoshiko Atsuta, Nobuhiro Kanemura, Takanori Teshima

**Affiliations:** 1 Department of Hematology and Infectious Disease, Gifu University Hospital, Gifu, Japan; 2 Department of Internal Medicine, Matsunami General Hospital, Gifu, Japan; 3 Department of Healthcare Economics and Quality Management, Graduate School of Medicine, Kyoto University, Kyoto, Japan; 4 Department of Hematology and Oncology, Graduate School of Medicine, Kyoto University, Kyoto, Japan; 5 Department of Hematology, Kyoto Katsura Hospital, Kyoto, Japan; 6 Department of Hematology, Tokyo Medical and Dental University, Tokyo, Japan; 7 Division of Hematology, Jichi Medical University Saitama Medical Center, Saitama, Japan; 8 Division of Emerging Medicine for Integrated Therapeutics (EMIT), Center for Molecular Medicine, Jichi Medical University, Shimotsuke, Japan; 9 Department of Hematology, Federation of National Public Service Personnel Mutual Aid Associations Toranomon Hospital, Tokyo, Japan; 10 Hematology Division, Tokyo Metropolitan Cancer and Infectious Diseases Center, Komagome Hospital, Tokyo, Japan; 11 Department of Hematopoietic Stem Cell Transplantation, National Cancer Center Hospital, Tokyo, Japan; 12 Department of Hematology, Hyogo Medical University Hospital, Hyogo, Japan; 13 Department of Hematology, Kanagawa Cancer Center, Kanagawa, Japan; 14 Department of Hematology, Japanese Red Cross Aichi Medical Center Nagoya Daiichi Hospital, Aichi, Japan; 15 Department of Hematology, Hokkaido University Hospital, Hokkaido, Japan; 16 Department of Hematology and Oncology, Okayama University Hospital, Okayama, Japan; 17 Department of Hematology and Oncology, Anjo Kosei Hospital, Aichi, Japan; 18 Department of Hematology, Hamanomachi Hospital, Fukuoka, Japan; 19 Department of Hematology/Oncology, Tokai University School of Medicine, Isehara, Japan; 20 Department of Hematology, Hiroshima Red Cross Hospital & Atomic-bomb Survivors Hospital, Hiroshima, Japan; 21 Central Japan Cord Blood Bank, Seto, Japan; 22 Technical Department, Japanese Red Cross Society Blood Service Headquarters, Tokyo, Japan; 23 Japanese Data Center for Hematopoietic Cell Transplantation, Nagakute, Japan; 24 Department of Registry Science for Transplant and Cellular Therapy, Aichi Medical University School of Medicine, Nagakute, Japan

**Keywords:** Epidemiology, Health care

## Abstract

The SARS-CoV-2 pandemic disrupted healthcare systems worldwide, particularly affecting hematopoietic stem cell transplantation (HSCT) activities. Understanding the impact of the SARS-CoV-2 pandemic on transplant practices, especially in Japan, where cord blood transplantation (CBT) is prevalent, is crucial. A total of 40,444 allogeneic HSCT cases in Japan between 2011 and 2021 were examined using an interrupted time series analysis to assess the impact of COVID-19 on CBT utilization. Following the SARS-CoV-2 pandemic, CBT cases demonstrated a significant increase (11.06 [95% confidence interval (CI): 1.87 to 20.25] cases per month), whereas bone marrow transplantation cases decreased, by 10.74 cases per month (95% CI, −19.84 to −1.63 cases per month). Total HSCT cases remained stable with a level change of 5.47 cases per month (95% CI, −10.07 to 21.01 cases per month) and a trend change of −1.11 cases per month (95% CI, −2.22 to 0.004 cases per month). The interrupted time series analysis showed significantly increased CBT cases in Japan, highlighting its crucial role as an alternative transplant source during the pandemic. CBT offset the impact of the decrease in bone marrow transplantation and contributed to the maintenance of HSCT activity in Japan during the unprecedented crisis.

## Introduction

The Coronavirus Disease 2019 (COVID-19) has profoundly impacted public health. The immediate threat is the morbidity and mortality associated with severe acute respiratory syndrome coronavirus-2 (SARS-CoV-2) [1], whereas the subtler threat lies in healthcare workers facing difficulties in providing routine medical care to vulnerable populations [2, 3], including patients with hematological disorders who were predisposed to COVID-19 due to their immunocompromised status [4, 5].

The emergence of COVID-19 affected hematopoietic stem cell transplantation (HSCT) activities worldwide. Patients undergoing conditioning or pre-treatment planning for HSCT are particularly vulnerable to the consequences of SARS-CoV-2 infection, given their immunocompromised state, and there is a risk that HSCT donors may become infected and unable to donate stem cells [6–8]. Our clinical practice shifted towards a more cautious approach, with increased emphasis on infection control measures and careful patient selection, to manage this unprecedented pandemic [3, 9]. Considering these challenges, organizations such as the Worldwide Network for Blood and Marrow Transplantation (WBMT), the Center for International Blood and Marrow Transplant Research (CIBMTR), and the European Society for Blood and Marrow Transplantation (EBMT) advocated for the cryopreservation of grafts and prioritizing domestic donors over international sources [10, 11]. Reports from the EU, Italy, France, and the global network noted substantial declines in HSCT donations and transplantations during the pandemic [12–14]. The international provision of unrelated donors decreased from 20,330 in 2019 to 19,623 in 2020, and the number of cord blood units shipped also decreased from 2851 to 2750 [4]. In contrast, in Japan, HSCT activities did not diminish in 2020 and were comparable to the previous two years [9]. Understanding the consequences and gaps of HSCT in Japan and other regions during the pandemic may have important clinical significance, particularly considering the availability of transplant donor sources.

One of the possible reasons for the maintenance of HSCT activities in Japan is the effective utilization of cord blood transplantation (CBT). CBT is the most widely used transplant donor source in Japan and among the highest globally [15]. Umbilical cord blood is a valuable stem cell source, and its cryopreserved stem cells are immediately available for urgent situations such as disasters, nuclear accidents, and pandemics, without exposure to SARS-CoV-2 [16]. The pandemic highlighted the added value of umbilical cord blood as an alternative donor source in emergencies. The safe and readily available options of CBT are anticipated to increase its usage during the pandemic.

The present study analyzed nationwide, representative, contemporary allogeneic HSCT registry data between 2011 and 2021 in Japan. The aim of the study was to explore the impact of COVID-19 on the choice of CBT in Japan. Interrupted time series (ITS) analyses were used to investigate the longitudinal effect of the SARS-CoV-2 pandemic.

## Materials and methods

### Study participants and design

All data were obtained from the Second-Generation Transplant Registry Unified Management Program (TRUMP 2) database, a web-based HSCT registry in Japan established to collect the clinical and prognostic data of patients and donors related to HSCT. TRUMP 2 is a nationwide survey of HSCT cases and a centralized tabulation and analysis project of the Japanese Data Center for Hematopoietic Cell Transplantation (JDCHCT) [17]. Patient consent was obtained before registration in TRUMP 2. This study was approved by the Data Management Committee of the JDCHCT and the Medical Review Board of Gifu University Graduate School of Medicine (#2023-305).

Allogeneic HSCT data were retrospectively collected from TRUMP 2 from January 1, 2011, to December 31, 2021. Allogeneic HSCT cases were analyzed, and the only exclusion criterion was non-consent to enrollment in the registry. The investigation conformed with the principles outlined in the Declaration of Helsinki and later amendments.

Data on baseline demographics were obtained from the TRUMP 2 database. The clinical summary data include patient demographics, transplantation date, diagnoses classified as the main trigger for transplantations, comorbidities and complications, and donor sources. The primary endpoint was the change in the number of HSCT cases per month. The secondary endpoint was the changes in the numbers of total allogeneic stem cell transplantation cases, bone marrow transplantation cases, and peripheral blood stem cell transplantation cases per month.

### Interrupted time series analyses for changes in case numbers

Single ITS analysis was used to study trends in CBT case numbers per month before and after the pandemic during the study period [18–20]. The detailed description of the ITS analysis is provided in the online Supplementary Methods.

### The SARS-CoV-2 pandemic in Japan

To establish the institutional context for the study, it is essential to provide a concise overview of the SARS-CoV-2 pandemic in Japan. Detailed information regarding the trends of COVID-19 infections and related official announcements in Japan during 2020 can be found in the Supplementary Methods.

### Sensitivity and exploratory analysis

Several sensitivity analyses were conducted. The detailed description of the sensitivity analyses is shown in the online Supplementary Methods.

To assess the robustness of the findings from the frequentist ITS analysis, a sensitivity analysis was performed using Bayesian time-series modeling. This approach allowed us to incorporate prior information and quantify uncertainty in the estimates. In contrast to confidence intervals, which estimate a range for a population parameter based on repeated sampling, Bayesian credible intervals represent a range of plausible values for the parameter, given the observed data and prior beliefs. In this analysis, 95% credible intervals, indicating a 95% probability that the true parameter lies within the calculated interval, given the data and the prior distribution used in the model, were calculated.

### Statistical analysis

Continuous variables are presented as median and interquartile range values. Mann–Whitney U tests were used to compare continuous variables between the two groups. Categorical data were assessed using chi-squared or Kruskal–Wallis tests, as appropriate. Significance was considered at *P* < 0.05 (two-tailed). Statistical analyses were conducted using R version 4.3.2 (R Foundation for Statistical Computing, Vienna, Austria) with the R packages ‘car’, ‘tsModel’, ‘nlme’, ‘CausalImpact’, and ‘tseries’.

## Results

### Overall study population

A total of 40,444 allogeneic HSCT cases (median age, 49.0 years; 23,998 (59.0%) males) were identified during the study period. Of them, HSCT cases before and after the SARS-CoV-2 pandemic accounted for 33,170 and 7274 cases, respectively. For CBT cases, there were 11,270 cases before and 2661 cases after the SARS-CoV-2 pandemic. The flow chart of the population before and after the SARS-CoV-2 pandemic in the present cohort is summarized in Supplementary Fig. 1. Patient demographics and donor sources are summarized in Table 1.

**Table 1:** Patients’ characteristics before and after the SARS-CoV-2 pandemic.

Baseline characteristic	Overall	Before/After pandemic	
		Before	After
	(*N* = 40,444)	(*n* = 33,170)	(*n* = 7274)
Age, y	49 (31, 60)	48 (30, 59)	51 (35, 61)
Sex, *n* (%)			
Female	16,430 (41)	13,469 (41)	2961 (41)
Male	23,998 (59)	19,686 (59)	4312 (59)
Performance status, *n* (%)			
0	18,884 (47)	15,229 (46)	3655 (50)
1	16,339 (41)	13,416 (41)	2923 (40)
2	3215 (8.0)	2775 (8.4)	440 (6.1)
3	1298 (3.2)	1145 (3.5)	153 (2.1)
4	517 (1.3)	460 (1.4)	57 (0.8)
Unknown	56 (0.1)	43 (0.1)	13 (0.2)
Number of HSCTs, *n* (%)			
0-1	32,415 (80)	26,504 (80)	5911 (81)
		−80	
≥2	8006 (20)	6641 (20)	1360 (19)
Donor source, *n* (%)			
Other	3 (<0.1)	3 (<0.1)	0 (0)
BMT	15,305 (38)	13,248 (40)	2057 (28)
BMT + PBSCT	71 (0.2)	60 (0.2)	11 (0.2)
PBSCT	11,134 (28)	8589 (26)	2545 (35)
CBT	13,931 (34)	11,270 (34)	2661 (37)
Donor type, *n* (%)			
Related	12,947 (32)	10,502 (32)	2445 (34)
Unrelated	27,497 (68)	22,668 (68)	4829 (66)
Conditioning, *n* (%)			
Reduced intensity	19,931 (49%)	16,326 (49%)	3605 (50%)
Myeloablative	20,341 (51%)	16,708 (51%)	3633 (50%)
Disease, *n* (%)			
EBV-associated disease	338 (0.8)	273 (0.8)	65 (0.9)
Else	39 (<0.1)	20 (<0.1)	19 (0.3)
Other leukemia	2100 (5.2)	1788 (5.4)	312 (4.3)
Lymphoid tumor	4528 (11)	3767 (11)	761 (10)
ALL	7126 (18)	5893 (18)	1233 (17)
AML	16,283 (40)	13,369 (40)	2914 (40)
Multiple myeloma	436 (1.1)	362 (1.1)	74 (1.0)
HPS and LCH	146 (0.4)	118 (0.4)	28 (0.4)
Primary immune deficiency syndrome	464 (1.1)	379 (1.1)	85 (1.2)
Solid cancer	358 (0.9)	305 (0.9)	53 (0.7)
MDS	5436 (13)	4313 (13)	1123 (15)
MPD	368 (0.9)	368 (1.1)	0 (0)
MPN	305 (0.8)	103 (0.3)	202 (2.8)
Autoimmune disease	14 (<0.1)	11 (<0.1)	3 (<0.1)
Congenital metabolic disorder	157 (0.4)	125 (0.4)	32 (0.4)
Aplastic anemia and hematopoietic disease	1461 (3.6)	1234 (3.7)	227 (3.1)
CML	885 (2.2)	742 (2.2)	143 (2.0)

*ALL* acute lymphoblastic leukemia, *AML* acute myeloid leukemia, *BMT* bone marrow transplantation, *CBT* cord blood transplantation, *CML* chronic myeloid disease, *EBV* Epstein-Barr virus, *HPS* hemophagocytic syndrome, *HSCT* hematopoietic stem cell transplantation, *LCH* Langerhans histiocytosis, *MDS* myelodysplastic syndrome, *MPD* myeloproliferative disease, *MPN* myeloproliferative neoplasm, *PBSCT* peripheral blood stem cell transplantation.

^a^Values are given as medians (interquartile range).

**Fig. 1:**
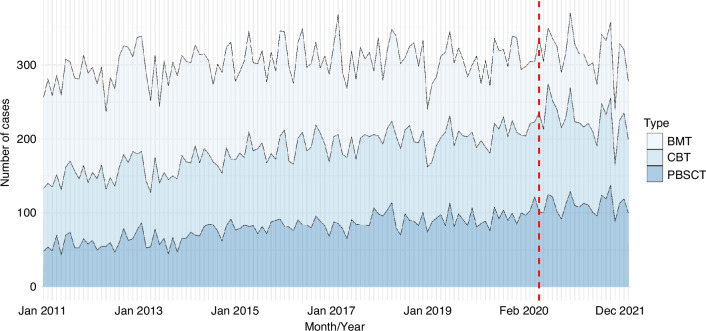
Stacked area graph of the absolute number of cases of donor sources among patients who underwent allogeneic stem cell transplants. The number of allogeneic stem cell transplant cases per month is plotted on the y-axis, and the date (year and month) on the x-axis. BMT bone marrow transplantation, CBT cord blood transplantation, PBSCT peripheral blood stem cell transplantation.

Temporal trends of donor sources in Japan are depicted in Fig. 1 and Supplementary Fig. 2. Of the HSCT cases before the pandemic, 11,270 (34%) CBTs, 13,248 (40%) BMTs, and 8589 (26%) PBSCTs were identified. Similarly, of the HSCT cases after the pandemic, 2661 (37.0%) CBTs, 2057 (28.0%) BMTs, and 2545 (35.0%) PBSCTs were identified. Figure 1 shows the absolute number of cases receiving allogeneic HSCT (BMT, CBT, and PBSCT) in a stacked area graph. Supplementary Fig. 2 demonstrates the proportional stacked area graph of donor sources for allogeneic HSCT cases. Of the patients who underwent allogeneic HSCT, the proportion of patients who received CBT and PBSCT increased substantially, whereas the absolute number of cases and the proportion of BMT cases decreased throughout the study period.

To further examine the immediate impact of the pandemic, monthly transplant volumes from February 2019 to January 2020 were compared with those from February 2020 to January 2021 (Supplementary Table 1 and Supplementary Fig. 3). This analysis showed substantial month-to-month variations in transplant activity, with CBT showing the largest year-over-year increase in February (34%) and BMT showing the largest decrease in July (−35.9%).

### Interrupted time series analyses for changes in CBT cases

**Fig. 2:**
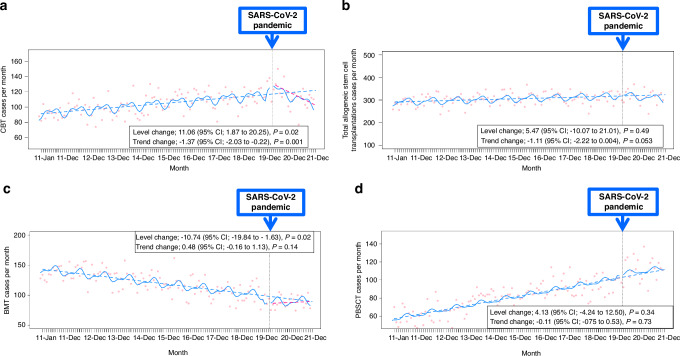
Interrupted time series analysis of the effect of the SARS-CoV-2 pandemic on hematopoietic stem cell transplantation. The figure displays interrupted time series analyses of monthly transplantation case numbers before and after the SARS-CoV-2 pandemic (vertical dotted line indicates February 2020). **a** Cord blood transplantation cases. **b** Total allogeneic stem cell transplantation cases. **c** Bone marrow transplantation cases. **d** Peripheral blood stem cell transplantation cases. Red dots represent observed monthly case numbers. Solid blue lines show predicted trends based on seasonally adjusted regression models. Dashed blue lines represent predicted linear trends post-pandemic. SARS-CoV-2: severe acute respiratory syndrome coronavirus-2.

Figure 2a shows the monthly number of CBT cases with the results of the single ITS time series analysis. ITS analysis adjusted for autocorrelation and non-linearity showed a significant increase in the level of CBT cases, by 11.06 [95% CI: 1.87–20.25] cases per month, with a significant change in the trend of CBT cases, after the SARS-CoV-2 pandemic (Fig. 2a).

### Exploratory analyses: interrupted time series analyses for changes in numbers of total allogeneic HSCT cases, BMT cases, and PBSCT cases

The exploratory analysis that included total allogeneic HSCT data before and after the SARS-CoV-2 pandemic showed that the total number of HSCT cases remained stable (level change 5.47 cases per month [95% CI, −10.07 to 21.01 cases per month] and trend change −1.11 cases per month [95% CI, −2.22 to 0.004 cases per month]) (Fig. 2b). An exploratory analysis including BMT cases only was also conducted. The ITS model, including the BMT cases only, verified the decreased level of BMT cases immediately after the pandemic (−10.74 cases per month [95% CI, −19.84 to −1.63 cases per month]) (Fig. 2c). The exploratory analysis with the ITS model by restricting the sample to PBSCT cases also showed that PBSCT activities were unaffected, with no trend and level changes in PBSCT cases during the post-pandemic period (level change 4.13 cases per month [95% CI, −4.24 to 12.50 cases per month] and trend change −0.11 cases per month [95% CI, −0.75 to 0.53 cases per month]) (Fig. 2d).

### Sensitivity analyses: interrupted time series analyses for changes in CBT cases with Bayesian time-series modeling and quasi-Poisson regression

**Fig. 3:**
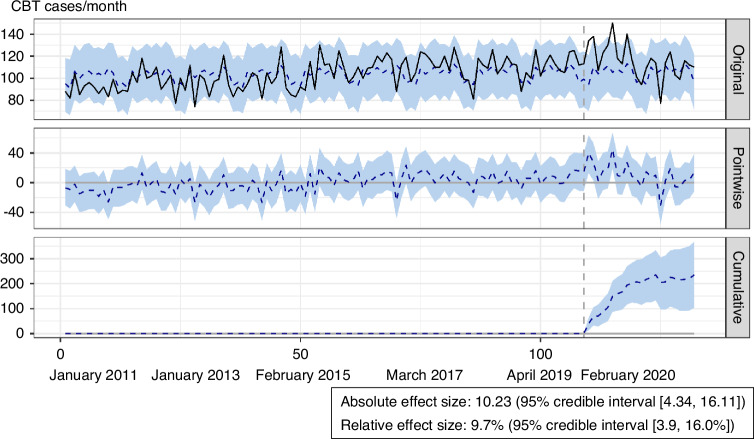
Bayesian causal inference modeling of the effect of the SARS-CoV-2 pandemic. Top panel: The original data (black) and the counterfactual estimate (blue dotted line with 95% credible interval). Second panel: The delta between the original data and the estimate showing the causal effect of every time point. Third panel: Cumulative monthly causal effects over time. CBT Cord Blood Transplantation, SARS-CoV-2 severe acute respiratory syndrome coronavirus-2.

Bayesian time-series modeling also confirmed that, even after controlling for the seasonal component, CBT cases increased, with an absolute effect size of 10.23 (95% credible interval [4.34, 16.11] cases) and a relative effect size of 9.7% (95% credible interval [3.9, 16.0%]) after the SARS-CoV-2 pandemic (Fig. 3). A segmented quasi-Poisson regression analysis of ITS was also performed to investigate the changes in CBT cases during the pandemic. The above sensitivity analysis also showed that CBT cases increased (level change 7.8% [95% CI, 0.7–14.3%]) soon after the pandemic (Fig. 3).

### Exploratory analyses (in children <18 years): interrupted time series analyses for changes in numbers of total allogeneic HSCT cases, BMT cases, and PBSCT cases

Of the total 40,444 patients, 5338 (13.2%) were children under 18 years of age. There were significant differences in stem cell source utilization between children and adults. BMT was more frequently used in children (49%) than in adults (36%), whereas PBSCT was more common in adults (29%) than in children (16%). CBT usage was similar between the two groups (34–35%) (Supplementary Table 2). These differences in stem cell source distribution between age groups remained consistent both before and after the onset of the SARS-CoV-2 pandemic (Supplementary Tables 2 and 3).

The ITS analysis did not show significant changes in donor source utilization for children before and after the pandemic (Supplementary Table 4). However, this analysis was limited by the relatively small number of pediatric cases (<100 per month), which may have reduced our ability to detect significant changes.

## Discussion

The present results demonstrated the following. First, after the SARS-CoV-2 pandemic, ITS analysis showed a significant increase in CBT cases per month. Though PBSCT cases did increase, the ITS analysis suggests that CBT played a critical role in offsetting the decrease in BMT, thereby maintaining overall HSCT activity. Third, total transplant activity did not decrease and was maintained throughout the pandemic in Japan. Last, the sensitivity analyses (segmented regression and Bayesian estimation) also confirmed the primary ITS analysis, supporting the robustness of the results of this study. These findings suggest that CBT functioned effectively as an alternative transplant source in the emergency situation in Japan.

The present findings, derived from ITS analysis, demonstrate that the SARS-CoV-2 pandemic had a significant impact on stem cell source utilization in Japan. Though pre-existing trends were present, the ITS analysis showed a significant level change in CBT cases following the onset of the pandemic, suggesting a specific effect of the pandemic on the adoption of CBT beyond existing trends. The observed increased use of CBT aligns with the supplementary analysis comparing monthly transplant volumes from February 2019 to January 2020 with those from February 2020 to January 2021, which showed a +34% peak in CBT cases. This emphasizes the importance of considering pre-existing trends and applying robust statistical methods to account for such trends. Since CBT is a major transplant donor source in Japan, with utilization comparable to BMT and PBSCT, this allowed the Japanese medical community to more quickly implement its usage than other countries [9, 12, 15].

The present findings confirmed that the SARS-CoV-2 pandemic led to a significant immediate increase in the number of CBT cases in Japan. CBT offers a variety of advantages over BMT: faster availability to transplant, tolerance of human leukocyte antigen mismatch, diminished donor attrition, and a lower risk of transmitting infections [16]. Driven by these evident CBT advantages, we presume that CBT usage increased soon after the pandemic occurred. The SARS-CoV-2 pandemic emphasized the importance of CBT as a critical and alternative transplant source, especially in emergencies such as nuclear accidents, natural disasters, or epidemics [14, 16].

The present study identified no detrimental impact of the SARS-CoV-2 pandemic on HSCT activities in Japan, whereas the number of BMT cases decreased rapidly during the pandemic. These results suggest that CBT functioned as an alternative donor source, which offset the decline in BMT cases in Japan. One can infer that the prompt procurement of CBT and the minimized risk of infection buffered the impact of the SARS-CoV-2 pandemic on HSCT and contributed to the maintenance of HSCT activities in Japan.

The sensitivity analyses using segmented regression and Bayesian estimation also confirmed the significant rise in cases of CBT during the pandemic. The results of the present study are also supported by the results of a previous study using TRUMP 2 data [9], in which the number of allogeneic transplants was maintained, and the 1-year overall survival after transplantation was also maintained. Previous observational studies found a decrease in HSCT cases in the EU, Italy, France, and the global network [12–14]. This discrepancy in findings was derived from differences in donor graft selection between Japan and other countries. Japan currently performs the largest number of CBT cases in the world and is the only country where the number of CBT cases is increasing [15]. In contrast, peripheral blood stem cells are the most common source for unrelated HSCT in Europe and the United States, and CBT is often used for transplantation in pediatrics. In Japan, the accumulation of experience in the number of CBT transplant systems [15] and the development of transplant networks facilitated swift and safe CBT during the pandemic.

Though there were significant changes in stem cell source utilization in the overall population, particularly an increase in CBT, these changes were not significant in the pediatric subgroup. This could be due to the smaller sample size of pediatric cases, resulting in reduced statistical power to detect changes. Alternatively, it may suggest that pediatric HSCT practices were less affected by the pandemic, possibly due to different risk-benefit considerations in children or the already high utilization of BMT in this population. Future studies with larger pediatric cohorts are needed to further elucidate the impact of the pandemic on HSCT practices in children.

There are several limitations of the present study. First, the impact of known or unknown factors, other than the SARS-CoV-2 pandemic of interest, should always be considered in an ITS analysis. If there are effects due to changes in pharmacotherapy or other unknown factors, it is difficult to exclude them in the study design if it is a single ITS. Another shortcoming of the study was that data on reasons for the stem cell source selection were not available in the TRUMP 2 database. Thus, the causes of the substantial reduction of BMT cases and the expansion of CBT in Japan are still open to debate. The present study was limited by the relatively short post-intervention period (ending in December 2021). Though this timeframe allowed the initial impact of the SARS-CoV-2 pandemic on HSCT practices to be captured, longer-term follow-up is needed to assess the sustained effects of the pandemic on transplant trends and patient outcomes. This analysis did not include data on transplant-related mortality (TRM) at day 100, a standard outcome variable in HSCT. Though the present study provides valuable insights into changes in transplant activity, future research should incorporate TRM day 100 to provide a more complete picture of the impact of the pandemic on patient outcomes. This would allow for a more thorough evaluation of the safety and efficacy of different transplant strategies during the pandemic. Future research should examine transplant activity beyond 2021 to determine whether the observed changes in stem cell source utilization persist or evolve over time. Further investigations are warranted to identify the cause of the maintained HSCT activity during the SARS-CoV-2 pandemic in Japan. In addition, the global impact of COVID-19 on burdens of HSCT activity, long-term adverse effects on morbidity and mortality in patients who underwent HSCT, and collateral damage to the healthcare system need to be addressed in cooperation with nationwide and worldwide research. It is a challenging task to reconcile the prevention of unjustified exposure of donors and recipients to COVID-19 infections with the provision of medical care to candidate HSCT patients in need of urgent treatment. We need to be ready for the next emergencies.

Though the absolute increase in PBSCT cases may appear larger when comparing pre- and post-pandemic periods, the ITS analysis demonstrated that CBT deviated significantly from its pre-pandemic trend, indicating a specific impact of the pandemic on CBT utilization beyond the existing trend.

## Supplementary information

Supplementary File

## Data Availability

The data of this study are not publicly available due to ethical restrictions that exceed the scope of the recipient/donor’s consent for research use in the registry. Data may be available from the corresponding author upon reasonable request and with permission of the JSTCT/JDCHCT.
