# Supplementary material for: The impact of the SARS-CoV-2 pandemic on umbilical cord blood transplantation in Japan: insights from an interrupted time series analysis
Source: Bone Marrow Transplant. 2025 Apr 17;60(7):964–70. doi: 10.1038/s41409-025-02588-0 (PMC12234360; doi:10.1038/s41409-025-02588-0)
Supplement: Supplementary file 1 — Supplementary File [file 41409_2025_2588_MOESM1_ESM.pdf]

Supplementary Methods

The COVID-19 pandemic in Japan

A concise overview of the COVID-19 pandemic in Japan is necessary to provide an institutional context for the study. The time period was determined, and three candidate “interruption” points were identified for interrupted time series (ITS) analysis: 1) February 2020, when the World Health Organization (WHO) declared the Public Health Emergency of International Concern (PHEIC); 2) March 2020 when the WHO declared the COVID-19 outbreak a global pandemic; and 3) April 2020, when a State of Emergency was declared in Japan. Anticipatory responses were taken into account. The model with the best Akaike’s Information Criteria (AIC) and Bayesian Information Criterion (BIC) values identified February 2020 as the optimal “interruption” point.

Interrupted time series analyses for changes in HSCT case numbers

The ITS design represents the temporal version of the continuous variable Z used for treatment assignment in a regression discontinuity design (RDD). The ITS is considered a robust quasi-experimental option when randomized designs are impractical or impossible. It has a control (baseline trend in the intervention group), allowing within-group comparisons. Though RDD and ITS share similarities, ITS, when repeatedly measuring outcomes for the same population, needs to address autocorrelation concerns. Autocorrelation signifies a correlation between outcomes in consecutive years in this study. In RDD, both sides of the Z cutoff compare different individuals, mitigating autocorrelation concerns. Conversely, ITS often tracks the same population, necessitating proper handling of autocorrelation. Thus, ITS, a special RDD type that accounts for autocorrelation, was used in the present research.

Monthly aggregated data are depicted in a time series format. The non-stationarity of the time series data was assessed with unit root testing procedures (augmented

Dickey-Puller test, Phillips-Perron test, and Kwiatkowski-Phillips-Schmidt-Shin). Residual autocorrelation was tested with the Ljung-Box test. Adjustment for autocorrelation was performed, and general linear regression models were developed to evaluate level and trend changes. Using generalized least squares models, autocorrelation terms for both moving average and autoregressive processes were incorporated. Autocorrelation and partial-autocorrelation functions for residuals were incorporated from ordinary least squares regression to determine the autoregressive-moving-average (p, q) model for the stationary series. The generalized least squares model was specified for error autocorrelation by including significant autoregressive parameters (p, q). Lag terms were identified using Durbin-Watson tests, autocorrelation, and partial autocorrelation plots. Assuming linearity in the pre-intervention period is often unrealistic; to address this, a harmonic term was incorporated into the model to account for non-linearity. In addition, seasonality was controlled by including harmonic terms (sines and cosines).

Sensitivity and exploratory analyses

Several exploratory and sensitivity analyses were conducted. First, ITS analysis was performed, not only on CBT cases, but also on the overall number of allogeneic HSCT cases, BMT cases, and PBSCT cases, to examine the impact of the COVID-19 pandemic. Second, a Bayesian structural time series model incorporating a seasonal element was used to estimate the causal effect, with the R package CausalImpact. Third, segmented regression analysis was performed to identify changes in the level of CBT cases during the study period with a quasi-Poisson distribution. A quasi-Poisson model was used to address the potential overdispersion of the data. Outcomes were analyzed by accounting for seasonality and secular trends before and after the COVID-19 pandemic. Seasonality was considered by including harmonic terms (sines and cosines). The validity of the model was also assessed

using correlograms and residual plots.

Supplementary Table 1. Transplant Cases

(Monthly comparison: between 2019-2020 and 2020-2021)

	period	month	BMT	PBSCT	CBSCT	total_cases
1	2019-2020	1	92	312	113	517
2	2020-2021	1	83	284	94	463
3	2019-2020	2	118	264	100	482
4	2020-2021	2	101	271	134	507
5	2019-2020	3	91	290	126	507
6	2020-2021	3	87	260	138	485
7	2019-2020	4	92	289	102	483
8	2020-2021	4	94	268	107	469
9	2019-2020	5	96	273	113	483
10	2020-2021	5	76	234	123	433
11	2019-2020	6	106	273	121	501
12	2020-2021	6	84	312	130	526
13	2019-2020	7	117	277	113	507
14	2020-2021	7	75	336	150	561
15	2019-2020	8	127	255	107	489
16	2020-2021	8	84	301	118	503
17	2019-2020	9	91	283	105	479
18	2020-2021	9	106	281	113	500
19	2019-2020	10	129	262	124	515
20	2020-2021	10	101	321	140	562
21	2019-2020	11	115	282	125	523
22	2020-2021	11	88	309	117	515
23	2019-2020	12	79	215	112	406
24	2020-2021	12	82	266	101	451

Supplementary Table 2. Patients' characteristics (adults versus children <18 years)

		Adults/Children	
**Baseline characteristic**	**Overall** (N = 40,444)	**Adults** (n = 35,106)	**Children** (n = 5,338)
Age, y	49 (31, 60)	52 (40, 61)	8 (1, 13)
Sex, n (%)			
Female	16,430 (41)	14,327 (41)	2,103 (39)
Male	23,998 (59)	20,769 (59)	3,229 (61)
Performance status, n (%)			
0	18,884 (47)	16,212 (46)	2,672 (50)
1	16,339 (41)	14,457 (41)	1,882 (35)
2	3,215 (8.0)	2,802 (8)	413 (8)
3	1,298 (3.2)	1,083 (3)	215 (4)
4	517 (1.3)	383 (1)	134 (3)
Unknown	56 (0.1)	39 (0.1)	17 (0.3)
Number of HSCTs, n (%)			
0-1	32,415 (80)	28,265 (81)	4,150 (78)
≥2	8006 (20)	6,816 (19)	1,180 (22)
Donor source, n (%)			
Other	3 (<0.1)	2 (<0.1)	1 (<0.1)
BMT	15,305 (38)	12,686 (36)	2,619 (49)
BMT + PBSCT	71 (0.2)	26 (0.1)	45 (0.8)
PBSCT	11,134 (28)	10,296 (29)	838 (16)
CBT	13,931 (34)	12,096 (35)	1,835 (34)
Donor type, n (%)			
Related	12,947 (32)	10,932 (31)	2,015 (38)
Unrelated	27,497 (68)	24,174 (69)	3,323 (62)
Conditioning, n (%)			
Reduced intensity	19,931 (49)	17,552 (50)	2,409 (45)
Myeloablative	20,341 (51)	17,426 (50)	2,915 (55)
Disease, n (%)			
EBV-associated disease	338 (0.8)	212 (0.6)	126 (2.4)
Else	39 (<0.1)	31 (0.1)	8 (0.1)
Other leukemia	2,100 (5.2)	2,049 (5.8)	51 (1.0)
Lymphoid tumor	4,528 (11)	4,322 (12)	206 (4)
ALL	7,126 (18)	5,602 (16)	1,524 (29)
AML	16,283 (40)	15,083 (43)	1,200 (23)
Multiple myeloma	436 (1.1)	435 (1.2)	1 (<0.1)
HPS and LCH	146 (0.4)	36 (0.1)	110 (2.1)
Primary immune deficiency	464 (1.1)	65 (0.2)	399 (7.5)

		Adults/Children	
Solid cancer	358 (0.9)	24 (0.1)	334 (6.3)
MDS	5,436 (13)	4,950 (14)	486 (9.1)
MPD	368 (0.9)	361 (1.0)	7 (0.1)
MPN	305 (0.8)	294 (0.8)	11 (0.2)
Autoimmune disease	14 (<0.1)	5 (<0.1)	9 (0.2)
Congenital metabolic disorder	157 (0.4)	28 (0.1)	129 (2.4)
Aplastic anemia and hematopoietic disease	1,461 (3.6)	786 (2.2)	675 (12.6)
CML	885 (2.2)	823 (2.3)	62 (1.2)

ALL, acute lymphoblastic leukemia; AML, acute myeloid leukemia; BMT, bone marrow transplantation; CBT, cord blood transplantation; CML, chronic myeloid disease; EBV, Epstein-Barr virus; HPS, hemophagocytic syndrome; HSCT, hematopoietic stem cell transplantation; LCH, Langerhans histiocytosis; MDS, myelodysplastic syndrome; MPD, myeloproliferative disease; MPN, myeloproliferative neoplasm; PBSCT, peripheral blood stem cell transplantation.

* Values are given as medians (interquartile range).

Supplementary Table 3. Patients' characteristics (children <18 years)

Baseline characteristic	Overall (N = 5,338)	Before/After pandemic<br>Before (n = 4,491)	After (n = 847)
Age, y	8 (4, 13)	8 (3, 13)	9 (4, 13)
Sex, n (%)			
Female	2,103 (39)	1,797 (40)	306 (36)
Male	3,229 (61)	2,689 (60)	540 (64)
Performance status, n (%)			
0	2,672 (50)	2,205 (49)	467 (55)
1	1,882 (35)	1,589 (35)	293 (35)
2	413 (7.7)	370 (8.2)	43 (5.1)
3	215 (4.0)	190 (4.2)	25 (3.0)
4	134 (2.5)	123 (2.7)	11 (1.3)
Unknown	17 (0.3)	12 (0.3)	5 (0.6)
Number of HSCTs, n (%)			
0-1	4,150 (78)	3,468 (77)	682 (81)
≥2	1,188 (22)	1,023 (23)	165 (19)
Donor source, n (%)			
Other	1 (<0.1)	1 (<0.1)	0 (0)
BMT	2,619 (49)	2,182 (49)	437 (52)
BMT + PBSCT	45 (0.8)	39 (0.9)	6 (0.7)
PBSCT	838 (16)	638 (15)	152 (18)
CBT	1,835 (34)	1583 (35)	252 (30)
Donor type, n (%)			
Related	2,015 (38)	1,680 (37)	335 (40)
Unrelated	3,323 (62)	2,811 (63)	512 (60)
Conditioning, n (%)			
Reduced intensity	2,409 (45)	2,020 (45)	389 (46)
Myeloablative	2,915 (55)	2,464 (55)	451 (54)
Disease, n (%)			
EBV-associated disease	126 (2.4)	108 (2.4)	18 (2.1)
Else	8 (0.1)	3 (0.1)	5 (0.6)
Other leukemia	51 (1.0)	42 (0.9)	9 (1.1)
Lymphoid tumor	206 (3.9)	171 (3.8)	35 (4.1)
ALL	1,524 (29)	1,311 (29)	213 (25)
AML	1,200 (23)	1,025 (23)	175 (21)
Multiple myeloma	1 (<0.1)	0 (0)	1 (0.1)
HPS and LCH	110 (2.1)	90 (2.0)	20 (2.4)
Primary immune deficiency	399 (7.5)	324 (7.2)	75 (8.9)

		Before/After pandemic	
Solid cancer	334 (6.3)	281 (6.3)	53 (6.3)
MDS	486 (9.1)	402 (9.0)	84 (9.9)
MPD	7 (0.1)	7 (0.2)	0 (0)
MPN	11 (0.2)	3 (0.1)	8 (0.9)
Autoimmune disease	9 (0.2)	8 (0.2)	1 (0.1)
Congenital metabolic disorder	129 (2.4)	104 (2.3)	25 (3.0)
Aplastic anemia and hematopoietic disease	675 (13)	552 (12)	123 (15)
CML	62 (1.2)	60(1.3)	2 (0.2)

ALL, acute lymphoblastic leukemia; AML, acute myeloid leukemia; BMT, bone marrow transplantation; CBT, cord blood transplantation; CML, chronic myeloid disease; EBV, Epstein-Barr virus; HPS, hemophagocytic syndrome; HSCT, hematopoietic stem cell transplantation; LCH, Langerhans histiocytosis; MDS, myelodysplastic syndrome; MPD, myeloproliferative disease; MPN, myeloproliferative neoplasm; PBSCT, peripheral blood stem cell transplantation.

* Values are given as medians (interquartile range).

Supplementary Table 4. Interrupted time series analysis (children <18 years)

Transplant types	Level change (95% CI)	P-value	Trend change (95% CI)	P-value
CBT	-1.1 (-4.5, 2.3)	0.53	-0.05 (-0.29, 0.20)	0.71
Total HSCT	1.9 (-4.0, 8.0)	0.53	-0.01 (-0.43, 0.42)	0.97
BMT	1.8 (-2.5, 6.1)	0.42	0.10 (-0.24, 0.41)	0.52
PBSCT	0.5 (-1.8, 2.8)	0.69	-0.06 (-0.23, 0.10)	0.45

BMT, bone marrow transplantation; CBT, cord blood transplantation; HSCT, hematopoietic stem cell transplantation; PBSCT, peripheral blood stem cell transplantation.

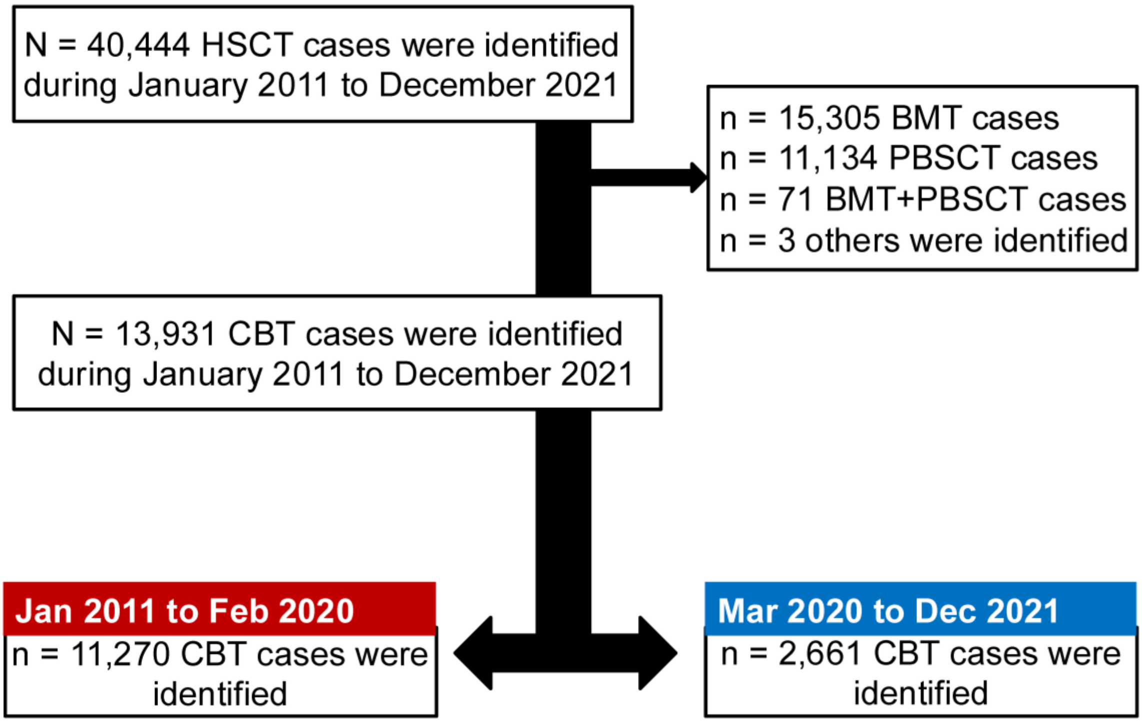

Supplementary Figure 1. Flow chart of the population that underwent HSCT between 2011 and 2021. Interrupted time series analyses for changes in CBT cases per month

BMT: bone marrow transplantation, CBT: cord blood transplantation, HSCT: hematopoietic stem cell transplantation.

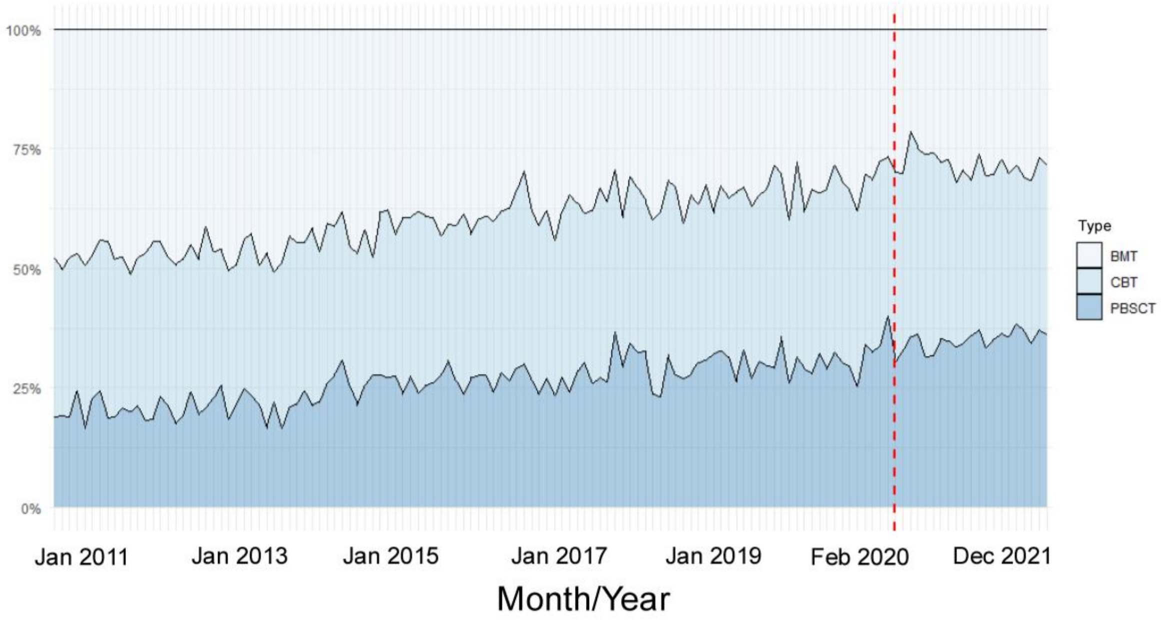

Supplementary Figure 2. Proportional stacked area graph of the proportion of donor sources for patients who underwent allogeneic stem cell transplants

The sum of each month is always equal to one hundred, and the value of each group is represented by percentages.

BMT: bone marrow transplantation, CBT: cord blood stem cell transplantation, PBSCT: peripheral blood stem cell transplantation.

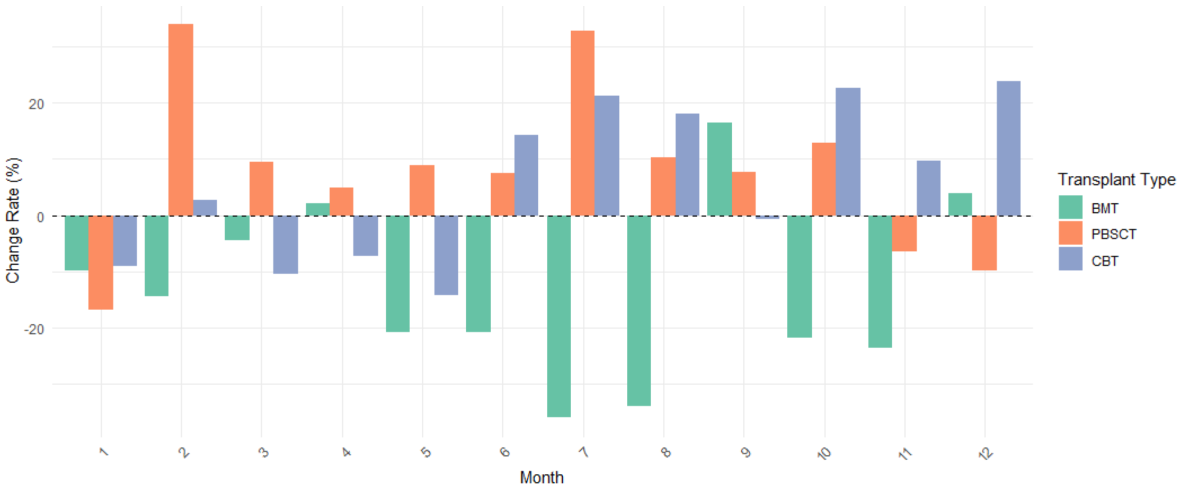

Supplementary Figure 3. Year-over-year percentage change in monthly transplant volume by stem cell source (BMT, CBSCT, PBSCT).

BMT: bone marrow transplantation, CBT: cord blood stem cell transplantation, PBSCT: peripheral blood stem cell transplantation.
